# Cortical thinning and white matter alterations in myotonic dystrophy type 2 over a 10-year period

**DOI:** 10.1007/s00415-025-13435-z

**Published:** 2025-10-15

**Authors:** Britta Krieger, Christiane Schneider-Gold, Erhan Genç, Onur Güntürkün, Christian Prehn, Barbara Bellenberg, Carsten Lukas

**Affiliations:** 1 Institute of Neuroradiology, St. Josef Hospital, Ruhr-University-Bochum, Gudrunstr. 56, 44791 Bochum, Germany; 2 Department of Neurology, St. Josef Hospital, Ruhr-University Bochum, Gudrunstr. 56, 44791 Bochum, Germany; 3 Department of Psychology and Neurosciences, Leibniz Research Centre for Working Environment and Human Factors (IfADo), Ardeystraße 67, 44139 Dortmund, Germany; 4 Department of Biopsychology, Institute of Cognitive Neuroscience, Faculty of Psychology, Ruhr University Bochum, Universitätsstr. 150, 44780 Bochum, Germany

**Keywords:** Myotonic dystrophy type 2, Brain MRI, Longitudinal, Cortical thickness, Fractional anisotropy

## Abstract

**Background:**

Cortical thinning and microstructural white matter alterations have been shown to occur in myotonic dystrophy type 2 (DM2). So far, longitudinal investigations are scarce and no significant differences of changes over 5 years were found between patients and healthy controls (HC). We hypothesized that DM2 might develop stronger brain alterations after long time periods of 10 years and that cortical thickness might be a representative biomarker for tracking cerebral changes in DM2.

**Methods:**

Ten DM2 patients and seven HC were included in this study who received two brain MRI examinations over 10 years. Brain images were segmented to obtain cortical thickness (CT) by use of CAT12. CT alterations were investigated with surface-based morphometry in CAT12. Diffusion tensor imaging data were pre-processed using MRtrix and fractional anisotropy (FA) maps were obtained by FSL’s dtifit, which were analyzed with tract-based-spatial statistics.

**Results:**

CT was significantly reduced over time in several brain areas in DM2 patients, but in only few regions in HC. DM2 patients showed decreased FA values in different brain regions, and larger differences from baseline to follow-up were obtained for DM2 patients than for HC.

**Conclusion:**

In this longitudinal study, distinct local patterns of cortical thinning and microstructural white matter alterations in DM2 over a 10-year period could be identified. These might serve as biomarkers for tracking long-term brain changes in DM2.

**Supplementary Information:**

The online version contains supplementary material available at 10.1007/s00415-025-13435-z.

## Introduction

Myotonic dystrophy type 2 (DM2), also known as proximal myotonic myopathy (PROMM), is an autosomal dominantly inherited multisystemic disorder characterized not only by myotonia, muscle weakness, and cataracts, but by CNS involvement. In Germany, a prevalence of 9 in 100,000 cases is suggested which corresponds to roughly 7200 patients [1]. DM2 primarily affects proximal muscles and results from a CCTG repeat expansion in the zinc finger protein 9 gene (ZNF9) [2, 3]. As a main pathophysiological mechanism, mRNA transcripts of CCTG repeat expansion are accumulated as mostly intranuclear ribonuclear foci interfering with splice factors and leading to miss splicing in various tissues. Despite longer repeat expansions in DM2 as compared to myotonic dystrophy type 1 (DM1), disease course is usually milder in DM2 than in DM1 [4, 5], with subsequently more studies focusing on DM1 than on DM2 in the past [6]. A recent review by Peric et al. (2021) summarized recent progress regarding cerebral involvement in DM2 [7]. They highlighted the cognitive impairment and clinical symptoms of DM2, such as memory and visuospatial impairment, fatigue, and pain. In our recent study, we provided evidence for cortical thinning occurring in both disease types appearing to be more widespread in DM1 than in DM2 [8]. Cortical thinning in DM2 seems to preferentially affect the temporal lobe in addition to the occipital lobe, which is common in both disease types [8].

As far as we know, only one study investigated longitudinal brain changes in DM2 [9]. They examined functional and structural cerebral changes in 16 DM1, 16 DM2 patients, and 17 healthy controls (HC) over 5 years. For DM2, they found no grey matter atrophy, stable white matter lesions (WML), but stronger white matter reduction and microstructural alterations at follow-up than at baseline. However, no significant differences of changes over 5 years were found between DM2 patients and HC.

Although previous results suggested only slowly progressive process or stability of cerebral abnormalities within 5 years [9], we aimed to investigate whether this also holds true over a longer time span. Therefore, we evaluated brain changes in DM2 over a 10-year period and additionally compared them to changes in HC. Since not only microstructural white matter changes but also cortical thinning were shown to be a feature of cerebral involvement in previous studies [6], the present analysis includes evaluation of longitudinal patterns of both cortical thinning and white matter integrity. We hypothesize that DM2 might develop more pronounced brain alterations after 10 years with cortical thickness probably representing a biomarker for tracking the extent of cerebral changes in DM2.

## Methods

### Participants

**Fig. 1:**
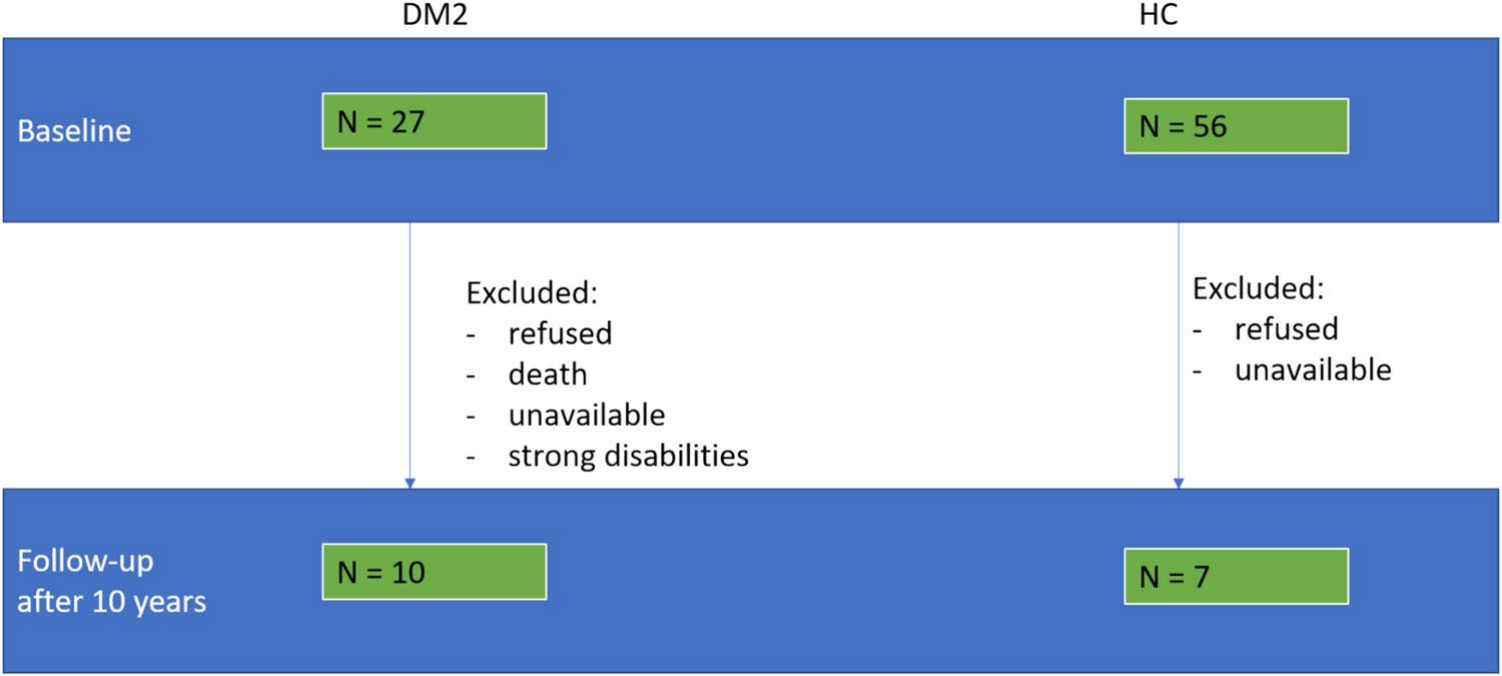
Numbers of included patients and healthy controls at baseline and follow-up and reasons for exclusion after 10 years

A total of ten DM2 patients and seven HC were included in this study who received two brain MRI examinations over 10 years between 2020 and 2022. Initially, 27 DM2 and 56 HC were scanned at baseline (BL), but follow-ups (FU) were not available for all of them due to refusal, unavailability, death, or strong disabilities (Fig. 1).

Images were acquired at the same scanner and MRI protocol as with the prospectively acquired participants. Patients older than 18 years were recruited in the neurological clinic if the diagnosis of DM2 was molecular-genetically proven. Exclusion criteria were strong dementing symptoms, contraindication for MRI acquisitions, taking psychopharmaca, heart pacemaker, renal insufficiency, pre-existing brain disorders, drug abuse, or severe psychological disorders. Patients with coincident cardiovascular, CNS- and psychiatric disorders, cancer or other severe illnesses were excluded from the follow-up study.

The study was approved by the local ethics committee of the Ruhr University Bochum (No. 20–7045, 06/10/2020), and all patients provided written informed consent prior to study participation.

### Magnetic resonance imaging

All MRI scans were acquired on a single 3 T Philips Achieva scanner with a 32-channel phased-array head coil. The MRI protocol included a high-resolution 3D T1-weighted (T1w) sequence (voxel size: 1 × 1x1 mm3, field of view: 240 × 240 × 180 mm^3^, TE: 4.6 ms, TR: 10 ms, flip angle: 8°, turbo factor: 164, acquisition time: 6 min), a 3D Fluid attenuated inversion recovery sequence (FLAIR, voxel size: 1 × 1x1 mm3, field of view: 240 × 240 × 170 mm^3^, TE: 286 ms, TR: 4800 ms, TI: 1650 ms, turbo factor: 182, acquisition time: 6.5 min), and diffusion-weighted imaging (DWI, voxel size: 2.5 × 2.5 × 2.5 mm^3^, field of view: 320 × 240 ×125 mm^3^, TE = 90 ms, TR = 7000 ms, 32 directions, b = 900 s/mm^2^, phase-encoding direction = PA) with one additional b0 image.

### MRI processing

White matter lesion segmentation was conducted using the longitudinal pipeline of LST (lesion segmentation toolbox) in SPM with FLAIR data and T1w as reference images [10]. Lesion filling was performed to prepare structural T1w images for further analyses. The CAT12 toolbox (version r1932 from 2022–01–13) [11] in SPM was then used to segment brain images and to obtain surface-based parameters including cortical thickness. For that purpose, default parameters were applied.

DTI data was pre-processed using MRtrix and FSL functions, which are mostly based on FSL pipelines [12]. These steps included denoising and removal of Gibb’s ringing artifacts. Since no additional acquisitions with reverse phase-encoding direction were performed, distortion correction was conducted using the recently developed approach synb0-disco, which generates an undistorted non-diffusion-weighted image from the structural T1w scan based on a deep learning approach [13]. Together with the real b0 image, the synthetic b0 image is then used as a target for distortion correction. The merged b0 images are then input to FSL’s topup function. Conventional eddy current and motion correction was then performed using FSL’s eddy. Diffusion tensor fitting was conducted using FSL’s dtifit to obtain fractional anisotropy (FA) maps.

### Clinical examination

All patients underwent a comprehensive neurological examination for clinical symptoms and CNS involvement (CSG). Daytime sleepiness was assessed by scoring according to the Epworth sleepiness scale (ESS). The ESS classifies daytime sleepiness into three stages: 0–10 as “no or moderate sleepiness”, 11–18 as “sleepy”, and 19–24 as “very sleepy” [14]. In addition, the presence of a restless-legs syndrome was examined by use of the Cambridge–Hopkins Questionnaire (short version 2) [15].

Furthermore, the degree of muscular impairment was categorized using the muscular impairment rating score (MIRS), which is a five-point rating scale developed to characterize the distal to proximal progression of muscular involvement in DM1. MIRS was adapted for DM2 patients as described previously [16]. For that, muscular impairment was graded to: grade 1 (no muscular impairment), 2 (minimal signs including myotonia, neck flexor weakness, no proximal weakness), 3 (proximal weakness but no distal weakness except for thumb and deep finger flexor weakness), 4 (proximal and distal weakness), and grade 5 (severe proximal and distal weakness). To overcome potential influences due to adaptation for DM2, the Modified Rankin Scale (MRS) [17] was also calculated as a more general and neutral score for patient’s disability. The MRS measures disability using five scores: 0 (no symptoms), 1 (no significant disability), 2 (slight disability), 3 (moderate disability), 4 (moderately severe disability), 5 (severe disability).

### Neuropsychological testing

Neuropsychological testing was performed by an experienced neuropsychologist (CP). The standardized neuropsychological test battery included tests for attention, alertness, memory, and executive functions. Specifically, selective attention was assessed by the d2-Test and the Go/no-Go test from the computerized Test for Attentional Performance (TAP). Divided attention and reaction ability (tonic and phasic alertness) were examined by TAP. Non-verbal intelligence was evaluated by the performance testing system LPS (Leistungsprüfungssystem) subtest 3 [18], flexibility of thinking by Regensburg verbal fluency test (RWT) [19], the verbal short-term memory by the Wechsler-Memory Scale (WMS–R) with digit span forward and backward [20], non-verbal visual working memory by WMS-R with block tapping forward and backward, verbal memory by Rey auditory verbal learning test (RAVLT), spatial visualization ability by LPS subtest 7, and general intelligence by the clock-drawing test and the Multiple-Choice Vocabulary Intelligence Test (MWT-B). Neuropsychological findings were classified into impaired or unimpaired according to normative data as provided by the specific test manuals. This was done by use of a threshold of one standard deviation below the normative mean. Neuropsychological test results were transformed into z scores to provide scores that are standardized with regard to age, gender, and education. For results of attention tests (d2-test, TAP subtests), that are largely independent from education, the 25 to 75 percentage range of the normative data was used as expectancy value for evaluation.

In addition, depression was scored using the Beck-Depression-Inventory-II (BDI-II), that graded values of 0 to 11 as normal, 11 to 19 as mild, 20 to 26 as moderate, and > 26 as severe depression [21].

### Statistical analysis

Global grey matter (GM), white matter (WM), and cerebrospinal fluid (CSF) volumes, and cortical thickness (CT) obtained from CAT12, total lesion volume (TLV), and global FA were compared between DM2 patients and HC at each time point by use of a linear mixed model analysis using R [22]. Age, sex, and TIV (not for TLV, CT, FA) were included as fixed effects, and subject as a random effect. Global volume changes were calculated as the difference between both time points and were compared between DM2 patients and HC using ANCOVA with age, sex, and TIV as covariates. Results were considered as significant at *p* < 0.05.

To assess local CT alterations between the two time points and between patients and healthy controls, surface-based morphometry (SBM) was performed in CAT12. Again, age and sex were included as covariates. Threshold-free cluster enhancement (TFCE) was applied with 5000 permutations, and FWE correction was used. Results were considered as significant for *p* < 0.05. Brain regions overlapping with significant clusters were printed by the toolbox’s atlas function using the Desikan–Killiany DK40 atlas. Difference maps between baseline and follow-up scans were created to visualize patterns of CT alterations.

Tract-based spatial statistics (TBSS) was conducted in FSL to evaluate group differences between the two time points and between patients and healthy controls [23, 24]. The pre-processed FA images were non-linearly registered to the 1 × 1 × 1 mm FMRIB58_FA standard space image. The resulting skeleton image was thresholded at 0.2 to remove the peripheral tract and grey matter [24]. Voxel wise statistics on the skeletonized data were conducted with age and sex as covariates, using FSL’s *randomize* with TFCE and 5000 permutations. FWE correction was applied to correct for multiple comparisons. Results were considered as significant for p < 0.05. Mean global FA values were extracted for each subject by use of *fslstats*.

Differences between neuropsychological test scores at baseline and follow-up were assessed by paired *t* tests using R [22]. Results were considered significant for *p* < 0.05.

## Results

### Demographic data

Demographic data of the study sample are summarized in Table 1. Patients were randomly included into the study which explains the disproportional male–female relation. All participants were Caucasian patients of European countries.

**Table 1:** Demographic data of patients and healthy controls for baseline (BL) and follow-up (FU) examination. *p* values were obtained from Kruskal–Wallis rank sum test or Pearson ‘s Chi-squared test

	DM2		HC	
	BL (*n* = 10)	FU (*n* = 10)	BL (*n* = 7)	FU (*n* = 7)
Age [years]^a^	49 (8)	58 (8)	42 (12)	52 (9)
Sex^b^				
Female	7 (70%)	7 (70%)	7 (100%)	7 (100%)
Male	3 (30%)	3 (30%)	0	0

^a^Mean (SD)

^b^Number (%)

### Global brain analyses

**Fig. 2:**
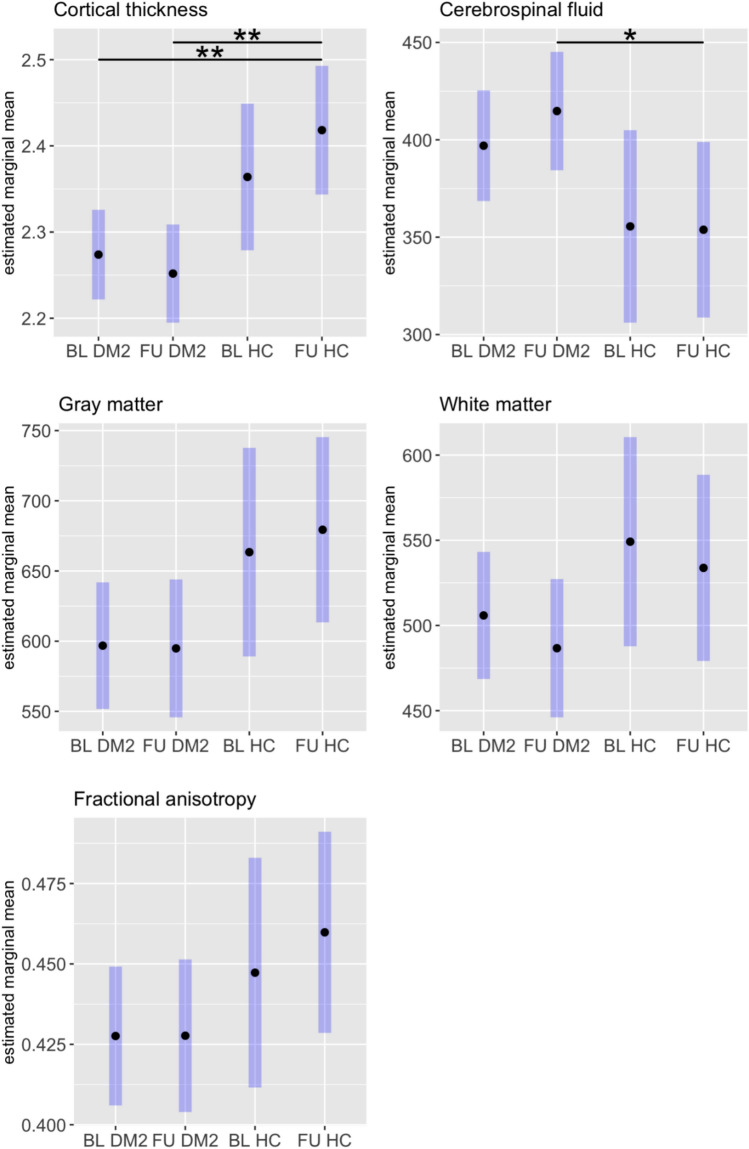
Longitudinal changes for global cortical thickness, cerebrospinal fluid, gray matter, white matter volumes and fractional anisotropy in myotonic dystrophy type 2 (DM2) patients and healthy controls (HC) from baseline (BL) to follow-up (FU) after 10 years. Black dots represent estimated marginal means from the model, and blue bars show the 95% confidence intervals

Our longitudinal analysis of global brain volumes and CT between DM2 patients and HC yielded significantly decreased CT in DM2 patients both at baseline (p = 0.006, 95% CI – 0.25, – 0.03) and at follow-up (p = 0.003, 95% CI – 6.12, – 1.56) compared to CT in HC at follow-up (Fig. 2). CSF volumes were further increased in DM2 patients at follow-up compared to HC at follow-up (*p* = 0.04, 95% CI 0.94, 7.52). Although not significant, we found a trend towards lower GM volumes in DM2 compared to HC both at baseline (*p* = 0.056, 95% CI – 94.6, 1.03) and at follow-up (*p* = 0.054, 95% CI – 102.6, 0.82) similar to the results for CT. No significant differences were found for global FA values and WM.

Absolute values for global brain volumes as well as CT and FA are summarized in Supplementary Table 1. On an individual patient level, all patients showed a decrease in GM, 90% showed a decrease in WM, and CSF increases were found in 90% of the patients. In addition, TLV was increased in seven of all ten patients. This descriptive brain imaging analysis is summarized in Supplementary Table 2.

To directly compare longitudinal change rates between both groups, volume or thickness changes were calculated as the differences between both time points (Table 2). Although DM2 showed larger changes in global GM volumes and CT than HC, these differences were not significant after adjustment for age and sex (Table 2).

**Table 2:** Longitudinal changes of gray matter (GM), white matter (WM), cerebrospinal fluid (CSF) volumes, cortical thickness, and fractional anisotropy (FA) in comparison between patients and healthy controls (HC)

Characteristic	DM2, n = 10^a^	HC, n= 7^a^	Difference^2^	95% CI^b,c^	*p* value^b^	Adjusted difference^4^	95% CI^b,c^	*p* value^b^
GM volume change	– 26 (20)	– 8 (10)	– 19	– 34, – 3.0	0.023	– 13	– 34, 7.2	0.2
WM volume change	– 18 (14)	– 14 (9)	– 3.8	– 16, 8.1	0.5	5	– 7.9, 18	0.4
CSF volume change	38 (26)	18 (9)	20	0.57, 39	0.045	5.5	– 18, 29	0.6
Cortical thickness change	– 0.07 (0.08)	0.01 (0.04)	– 0.08	– 0.14, – 0.02	0.015	– 0.07	– 0.15, 0.02	0.11
FA change	– 0.014 (0.024)	– 0.001 (0.01)	– 0.01	– 0.03, 0.01	0.2	0.0	– 0.03, 0.02	0.7

^a^Mean (SD)

^b^Welch two sample *t* test

^c^CI = confidence interval

^d^ANCOVA with age and sex as covariates

### Regional analyses

**Fig. 3:**
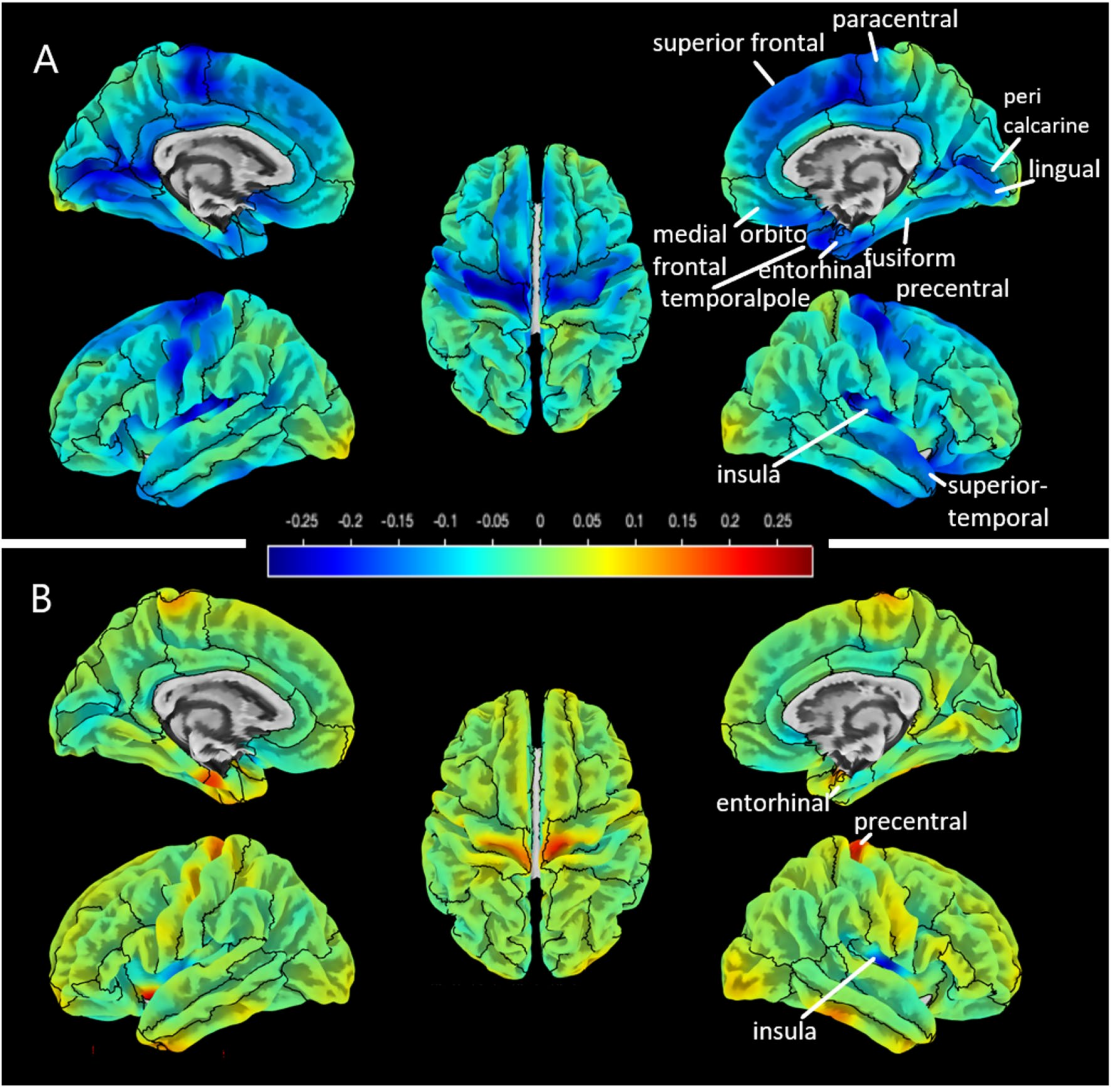
Difference maps for visualization of cortical thickness changes in myotonic dystrophy type 2 (DM2) patients (**A**) and healthy controls (**B**) over 10 years. Regions of interests that showed significant cortical thinning were included from Desikan–Kiliany atlas. The color scale ranges from – 0.25 mm decrease (blue) to 0.25 mm increase (red)

CT was significantly reduced in several brain areas in DM2 patients during follow-up: superior frontal, pre- and paracentral, pericalcarine, lingual, fusiform, entorhinal, medial orbitofrontal, superior temporal gyri, temporal pole, and insula. Most regions showed a decrease of – 0.05 to – 0.25 mm (as indicated in blue colors). In HC, the entorhinal, precentral gyri, and the insula showed significant reductions of CT which were smaller than in the patient group. This is visualized on difference maps (Fig. 3). No significant region was obtained when comparing the CT difference maps between patients and HC.

**Fig. 4:**
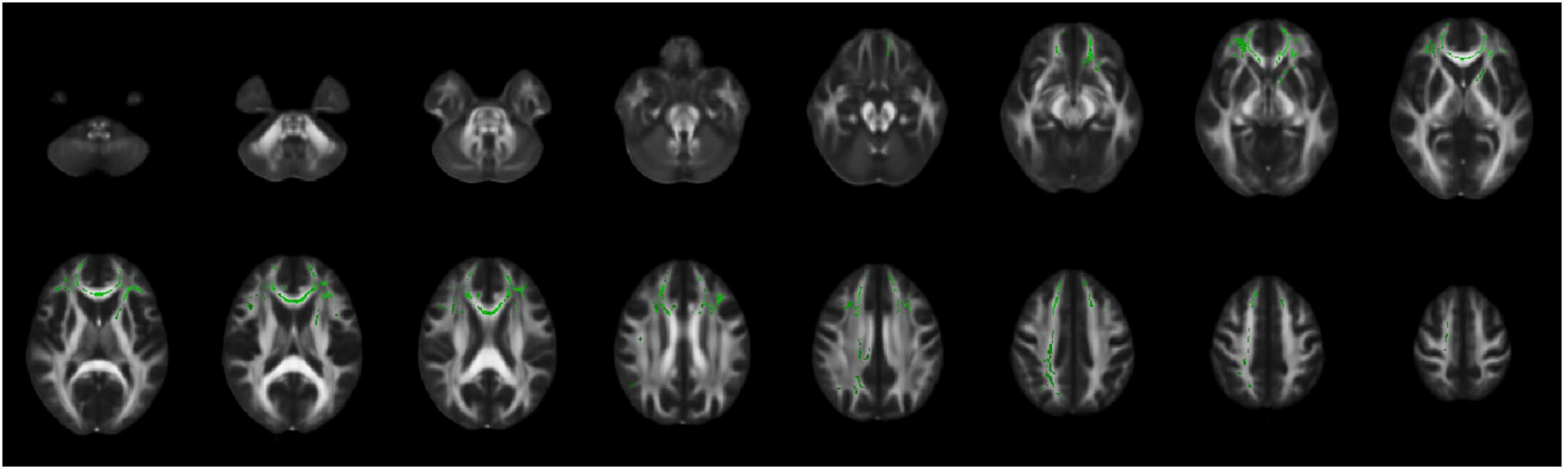
Decrease of fractional anisotropy within the myotonic dystrophy type 2 (DM2) patients over 10 years as indicated in green voxels on a FMRIB58 FA template in standard space

DM2 patients showed decreased FA during the 10-year follow-up period in different white matter regions: body and genu of corpus callosum, corticospinal tract (right), superior thalamic radiation (right), superior longitudinal fascicles 1 and 3, frontal aslant tract (right and left), inferior fronto-occipital fascicles (right and left), anterior thalamic radiation (right and left) (Fig. 4).

**Fig. 5:**
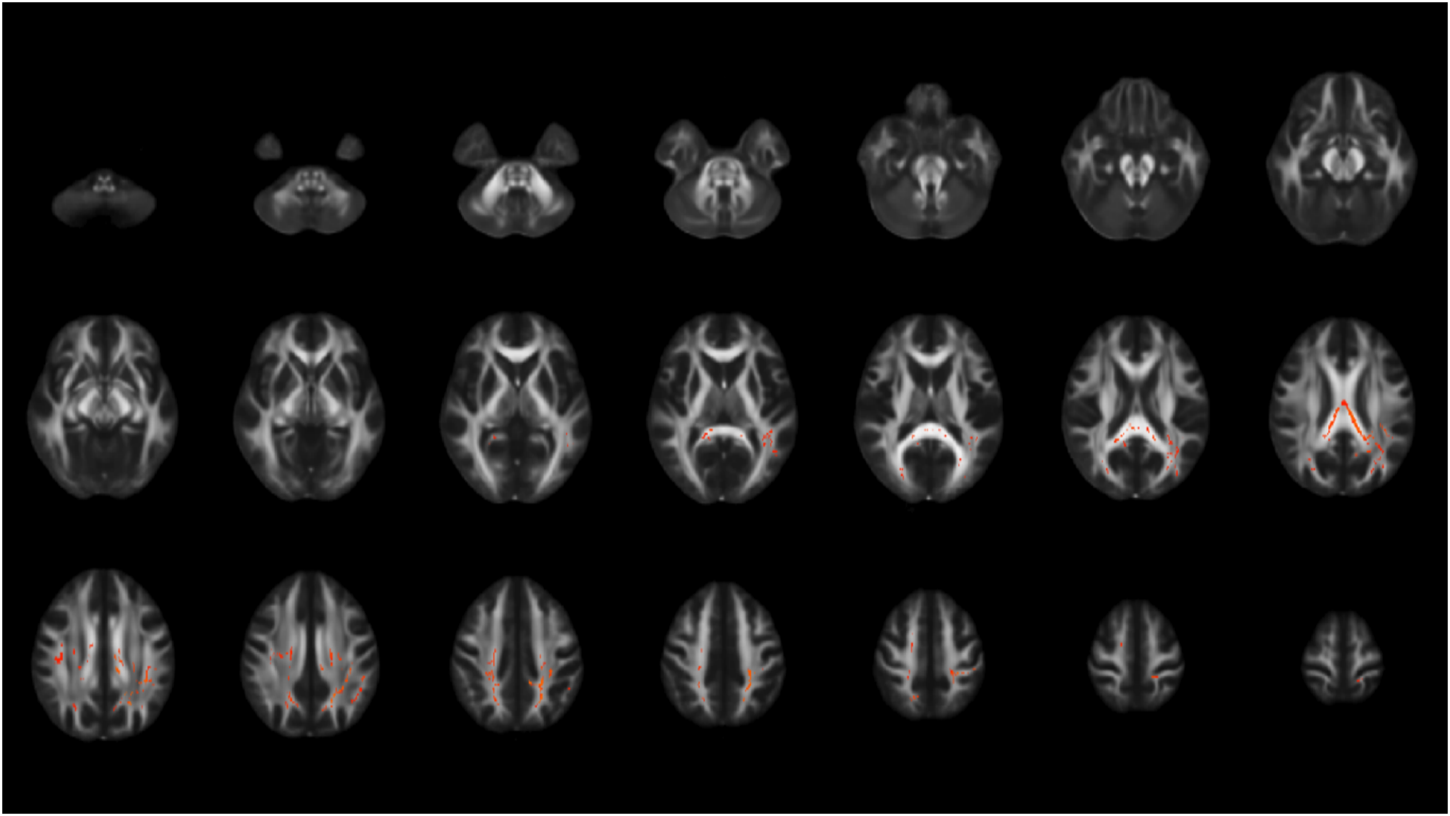
Comparison between longitudinal difference maps of FA between myotonic dystrophy type 2 (DM2) patients and healthy controls (HC). FA difference maps were calculated for DM2 and HC between baseline and follow-up and were statistically compared. The red voxels on a FMRIB58 FA template in standard space show regions with significantly larger FA decrease over time in DM2 compared to HC

Comparisons between difference maps of FA from baseline to follow-up between DM2 and HC yielded larger values in the splenium of corpus callosum, the superior longitudinal fascicles 1 and 2 (right and left), the forceps major, and left arcuate fascicles (Fig. 5).

### Clinical data and neuropsychological results

During a time period of 10 years, DM2 patients showed increased muscle impairment at follow-up evaluated by the grades from adapted MIRS (difference BL-FU: – 1.5, 95% CI = – 2.2, – 0.8, *p* < 0.001). BDI scores did not differ between baseline and follow-up examination (*p* = 0.7, 95% CI = – 7.9, 5.7). Results from ESS were not different between both time points (*p* = 0.2, 95% CI = – 1.1, 5.6). Furthermore, few significant differences between neuropsychological test results were observed: the number of omissions in TAP (divided attention) were decreased (difference BL-FU = 19, p = 0.046, 95% CI = 0.52, 37), whereas the reaction time in TAP (divided attention) showed a trend towards increase (difference BL-FU = −27, *p* = 0.078, 95% CI = – 58, 4.8). In the WMS-R with digit span forward, the digits slightly decreased (difference BL-FU = 0.7, *p* = 0.045, 95% CI = 0.02, 1.4). Restless legs syndrome was consistently present in four patients. Results from clinical and neuropsychological assessments are summarized in Table 3.

**Table 3:** Results from clinical and neuropsychological assessments as baseline (BL) and follow-up (FU)

	BL	FU	*p* value
Clinical assessment			
MIRS	2.0 (1.0,2.0)	4.0 (3.0,4.0)	< 0.001
ESS	5.5 (3.0,7.8)	4.0 (1.0,6.0)	0.2
Restless legs syndrome	4 (40%)	4 (40%)	> 0.9
BDI	16 (5, 23)	15 (10, 24)	0.7
Neuropsychological assessment			
Selective attention (d2, KL)	44 (29)	50 (27)	> 0.9
Alertness			
Tonic	32 (23)	44 (24)	0.4
Phasic	34 (25)	49 (30)	0.3
Go/no-go reaction time	43(29)	51 (29)	0.3
TAP divided attention			
Reaction time	26 (15)	50 (22)	0.078
Missed events	40 (22)	25 (19)	0.046
Non-verbal intellectual performance (LPS 3)	62 (23)	69 (19)	0.2
Time	53.8 (7.1)	55.6 (6.1)	0.3
Verbal fluency test RWT			
Fluency	51 (29)	45 (28)	0.7
Category change	36 (26)	42 (34)	0.3
Digit span WMS-R			
Forward (*n* digits)	6.8 (0.92)	6.1 (0.57)	0.045
Backward (*n* digits)	4.7 (1.06)	5.2 (1.23)	0.1
Block tapping WMS-R			
Forward	38 (34)	26 (21)	0.086
Backward	37 (32)	36 (27)	0.7
LPS 7	54 (22)	52 (28)	0.7
Time	54 (11)	50 (9)	0.2
Clock test (raw points)			0.3
1	9 (90%)	7 (78%)	
3	1 (10%)	2 (22%)	

Unless not otherwise specified, neuropsychological test results were derived from percentile ranks. Values are depicted as number (%), mean (SD) or median (IQR). *p* values were derived from paired *t* tests

*ESS * Epworth sleepiness scale, *MIRS*  muscular impairment rating score, *KL* concentration performance value

## Discussion

In this longitudinal study, new insights could be achieved regarding volumetric and microstructural brain changes in myotonic dystrophy type 2 over 10 years. First, both global and regional cortical thinning appeared to be able to track brain changes in DM2 and seemed to be more sensitive than GM or WM volume changes. Thus, compared to HC, we found that global CT was decreased at follow-up in DM2 patients. On regional level, strikingly more brain regions were affected by cortical thinning in our patient group than in HC, especially the temporal lobes, the superior frontal cortex, pre- and paracentral gyri, and the occipital lobes. Since to date only one study investigated longitudinal brain changes in DM2 patients [9], comparison with previous results is limited here. The study by Gliem et al. did not find any GM decrease in DM2 over a time period of 5 years. On the one hand, this might provide evidence that CT represents a more sensitive marker to cerebral changes than GM morphometry, which might also be suggested from our global longitudinal analysis that showed no significant global GM reduction. On the other hand, in the previous long-term follow-up study, the time period of 5.5 years might have been too short to observe any changes that occur in the cortex.

In our recent cross-sectional study, a focus of cortical thinning in the temporal lobe was found for DM2 patients, and the cuneus, precuneus, and lingual gyrus showed most pronounced deviations from HC [8]. In the present longitudinal study, not only the temporal lobe was affected by cortical thinning over 10 years, but also large parts of the superior frontal cortex, the pre- and paracentral gyri. Deficits in the precentral gyrus (primary motor cortex) might be associated with muscle weakness, which seemed to be worsened over time in our DM2 group. In addition, the occipital lobe, which were shown to be reduced in both DM1 and DM2 patients [8], also showed enhanced longitudinal cortical thinning with a main focus on the lingual gyrus. The lingual gyrus has been shown to play a role in visual processes, including the visual memory, which might be associated with our observed increased reaction time in the divided attention tests.

Evidence for GM involvement in DM2 in different brain regions has been provided by other previous cross-sectional studies which investigated GM loss by voxel-based morphometry techniques, but not cortical thickness. In an early study by Weber et al., GM loss was found in frontal and parietal lobes, hippocampi, and thalami [25]. Furthermore, a previous study from our own group showed clusters of GM atrophy in the cuneus, temporal, insular, and left medial frontal regions and in the amygdala in DM2 [16]. Plausibly, the results of our long-term follow-up study showed a progressive loss of CT especially in those areas that have been shown to be prone to GM atrophy or reduced CT in cross-sectional comparisons with HC. Taken together, our study suggested that cortical thinning is sensitive to tracking longitudinal changes in the brain over 10 years, and might provide complementary information compared to gross morphometric GM changes.

Second, microstructural WM damage seemed to be worsened after 10 years in DM2. Several white matter tracts were affected by reduced FA, and at the same time, differences between baseline and follow-up appeared to be larger in patients compared to controls, indicating stronger neurodegeneration. In the longitudinal DTI analysis by Gliem et al., no differences in changes over 5 to 6 years were found between DM2 patients and controls [9]. However, in patients, they observed additional FA reduction in regions, that were not decreased at baseline. In particular, the corpus callosum, the left inferior longitudinal and inferior fronto-occipital fascicles showed FA reductions. Compared to our results, we also found decreased FA over 10 years in the corpus callosum, the bilateral inferior fronto-occipital fascicles, and in the longitudinal fascicles, but in the superior instead of the inferior part. The overlaps in significant regions between both studies showed confirmatory results and the additional regions that appeared significantly decreased in our study might represent regions that developed microstructural tissue damage over longer time periods than 5 years.

Third, our patients showed slightly increasing deficits in divided attention reaction times as well as in verbal short-term memory. Compared to the longitudinal study from Gliem et al., they only found significant difference in changes over time visuoconstructive abilities measured by the Block Design Test [9]. Since different test batteries were used in our study, direct comparison is limited. However, visuoconstructive deficits would be in line with damage to the lingual gyrus, which showed enhanced cortical thinning in our study. For the interpretation of neuropsychological re-tests, it has to be considered that potential learning effects might lead to limited reliability of the results, which in turn would lead to an underestimation of cognitive deficits and to misjudgment of therapy successes. However, learning effects would not be expected after 10 years as it has been already described that these effects occur within intervals of 7 years [26].

Overall, we observed only subtle neuropsychological changes, which do not directly reflect the degree of MRI changes. On the one hand, this might be—at first—a positive message for DM2 patients and may indicate the existence of some compensatory mechanisms preserving cognitive capacities. Further research on such compensatory mechanisms could be very valuable. On the other hand, our findings still indicate ongoing brain pathology and could serve as a biomarker in case preventive therapeutic or neuroprotective approaches are available.

Some limitations must be considered for interpretation of our results. First, this study might be based in absolute terms on a small sample of patients, although the number of patients with DM2 is higher compared to the other studies. DM2 is a rare disease (prevalence of 9 in 100,000), which limits the possibilities to recruit patients that correspond to our strict inclusion criteria. Re-evaluation of patients and HC after 10 years was further challenged by the participants’ availability, the lack of capability to travel to the department or to stay in the scanner for back pain and other issues, the exclusion of patients that had experienced any cardiovascular event in between, and hardware changes to the MRI scanner that made it impossible to replicate the baseline and follow-up examinations with the same settings from a certain time point. Second, we were not able to recruit male controls. Since the patient group was predominantly female, this does not seem to be a major bias. In addition, the male patients included into this study did not show any particular imaging or clinical features. Studies on the difference of CNS findings in male and female patients in myotonic dystrophies and in HC during aging are missing so far. Thus, we cannot totally exclude a bias, but due to the mainly female patient group, a major bias seems to be unlikely. Third, no common method of distortion correction could be done with DTI raw data, because older scans were acquired without additional b0 with opposite phase-encoding direction. This method was developed in the past years so that it was not common 8–10 years ago when our first dataset was acquired. Instead, we used the recently developed synb0-disco approach as described in the methods. Thus, all data were handled the same and methods were not mixed. Fourth, no associations between clinical results and MRI parameters were examined since it was not the focus of our study and requires multiple comparisons that were limited by the small sample size.

### Conclusion

The present study underscores the relevance of longitudinal cerebral alterations for the interpretation of disease pathology in myotonic dystrophy type 2. Changes of microstructural white matter integrity and cortical thinning should be considered as potential biomarkers to monitor cerebral involvement in the disease course in DM2. In the future, larger and multi-centric, long-term follow-up studies are required to investigate the relationships between temporal cerebral changes and clinical disease manifestations, and to develop effective treatment strategies for DM2.

## Supplementary Information

Below is the link to the electronic supplementary material.

Supplementary file1 (DOCX 18 KB)

## Data Availability

The datasets for this article are not publicly available due to concerns regarding participant anonymity.

## Abbreviations

CNS: Central nervous system
CSF: Cerebrospinal fluid
CT: Cortical thickness
DM: Myotonic dystrophy
ESS: Epworth sleepiness scale
FA: Fractional anisotropy
FLAIR: Fluid attenuated inversion recovery sequence
GM: Gray matter
HC: Healthy control
MIRS: Muscular impairment rating score
MRI: Magnetic resonance imaging
MRS: Modified Rankin Scale
SBM: Surface-based morphometry
TBSS: Tract-based spatial statistics
TFCE: Threshold-free cluster enhancement
TLV: Total lesion volume
WM: White matter
