# Supplementary material for: Cortical thinning and white matter alterations in myotonic dystrophy type 2 over a 10-year period
Source: J Neurol. 2025 Oct 15;272(10):695. doi: 10.1007/s00415-025-13435-z (PMC12528299; doi:10.1007/s00415-025-13435-z)
Supplement: Supplementary file 1 — Supplementary file1 (DOCX 18 KB) [file 415_2025_13435_MOESM1_ESM.docx]

**Supplementary Information**

**Cortical thinning and white matter alterations in myotonic dystrophy type 2 over a ten years period**

**Authors**: Britta Krieger^a^, Christiane Schneider-Gold^b^, Erhan Genç^c^, Onur Güntürkün^d^, Christian Prehn^b^, Barbara Bellenberg^a^, Carsten Lukas^a^

**Affiliations:**

^a^ Institute for Neuroradiology, St. Josef Hospital, Ruhr-University-Bochum, Gudrunstr. 56, 44791 Bochum, Germany

^b^ Department of Neurology, St. Josef Hospital, Ruhr-University Bochum, Gudrunstr. 56, 44791 Bochum, Germany

^c^ Department of Psychology and Neurosciences, Leibniz Research Centre for Working Environment and Human Factors (IfADo), Ardeystraße 67, 44139 Dortmund, Germany

^d^ Department of Biopsychology, Institute of Cognitive Neuroscience, Faculty of Psychology, Ruhr University Bochum, Universitätsstr. 150, 44780 Bochum, Germany

**Corresponding author:** Britta Krieger, email address: [britta.krieger@rub.de](mailto:britta.krieger@rub.de)

Supplementary Table 1 Total intracranial volumes (TIV), gray matter (GM), white matter (WM), and cerebrospinal fluid (CSF) volumes, cortical thickness, total lesion volumes (TLV), and fractional anisotropy (FA) of myotonic dystrophy type 2 (DM2) patients and healthy controls (HC) at baseline and follow-up. ANCOVA was conducted by use of age, sex, and TIV (not for TIV, CT, and TLV) as covariates to compare DM2 and HC at baseline and follow-up.

|  | **BL ^1^** | | **FU ^1^** | | **Adjusted difference ^2^** | **95 % CI ^2,3^** | **p-value ^2^** |
| --- | --- | --- | --- | --- | --- | --- | --- |
|  |  |  |  |  | **BL / FU** | **BL / FU** | **BL / FU** |
|  | **DM2** | **HC** | **DM2** | **HC** |  |  |  |
|  | **N = 10** | **N = 7** | **N = 10** | **N = 7** |  |  |  |
| TIV [ml] | 1442 (181) | 1422 (119) | 1436 (177) | 1418 (117) | -78 / -73 | -214,59 / -207,60 | 0.2 / 0.3 |
| GM [ml] | 581 (79) | 635 (62) | 554 (83) | 627 (60) | -39 / -46 | -73,-5.1 /-90,-2.4 | 0.028 / 0.04 |
| WM [ml] | 484 (67) | 494 (56) | 466 (65) | 480 (51) | -10 / -5.2 | -36,15 / -29,19 | 0.4 / 0.6 |
| CSF [ml] | 377 (54) | 292 (36) | 416 (70) | 311 (38) | 49 / 51 | 10,89 / 2.1,100 | 0.018 / 0.042 |
| CT [mm] | 2.28 (0.08) | 2.41 (0.04) | 2.21 (0.12) | 2.41 (0.04) | -0.10 / -0.16 | -0.17,-0.03 / -0.27,-0.05 | 0.008 / 0.007 |
| TLV [ml] | 16 (29) | NA | 19 (28) | NA |  |  |  |
| FA | 0.43 (0.04) | 0.449 (0.013) | 0.41 (0.04) | 0.447 | -0.02 / -0.0.3 | -0.06,0.02 / -0.06,0.00 | 0.2 / 0.086 |
| ^1^ Mean (SD); n (%), ^2^ ANCOVA (DM2 vs. HC), ^3^ CI = Confidence Interval | | | | | | | |

Supplementary Table 2 Individual descriptive brain imaging analysis which summarises gray matter (GM), white matter (WM), cerebrospinal fluid (CSF), and total lesion volume (TLV) for each of the ten DM2 patients at baseline (BL) and follow-up (FU). Decreases at FU are highlighted in red and increases in green.

|  | **GM [ml]** | | **WM [ml]** | | **CSF [ml]** | | **TLV [ml]** | |
| --- | --- | --- | --- | --- | --- | --- | --- | --- |
|  | **BL** | **FU** | **BL** | **FU** | **BL** | **FU** | **BL** | **FU** |
| patient1 | 536 | 519 | 469 | 438 | 353 | 394 | 1.624 | 2.047 |
| patient2 | 500 | 491 | 473 | 433 | 372 | 421 | 94.06 | 88.2 |
| patient3 | 627 | 618 | 470 | 467 | 383 | 380 | 0.656 | 0.615 |
| patient4 | 531 | 499 | 420 | 416 | 356 | 388 | 14.041 | 25.238 |
| patient5 | 684 | 668 | 567 | 535 | 439 | 472 | 0.447 | 0.335 |
| patient6 | 732 | 708 | 604 | 588 | 402 | 431 | 1.295 | 1.298 |
| patient7 | 497 | 460 | 377 | 355 | 324 | 382 | 6.682 | 9.841 |
| patient8 | 577 | 554 | 503 | 486 | 358 | 394 | 0.894 | 1.433 |
| patient9 | 584 | 508 | 512 | 493 | 484 | 577 | 11.875 | 12.665 |
| patient10 | 533 | 512 | 440 | 444 | 297 | 312 | 29.939 | 44.283 |
